# Componeer as an aesthetic treatment option for anterior teeth: a case report

**DOI:** 10.1186/s12903-024-04081-4

**Published:** 2024-03-21

**Authors:** Irmaleny Irmaleny, Opik Taofik Hidayat, Raden Ajeng Pritasya Handayani

**Affiliations:** Department of Conservative Dentistry, Faculty of Dentistry, Universitas Padjadjaran, Bandung, Indonesia

**Keywords:** Componeer, Direct veneer, Aesthetic treatment, Composite veneer, Peg shape, Tooth anomalies, Prefabricated veneers, Anterior restoration, Minimally invasive

## Abstract

**Introduction:**

Structural abnormalities or anomalies in the anterior teeth, also known as the aesthetic zone, are an important problem for patients and a challenge for dentists. Structural abnormalities or tooth anomalies can change in color, shape, and function. Most dentists prefer minimally invasive aesthetic treatment. One of the aesthetic treatment options for anterior teeth is veneers. Veneer is a restoration that covers the labial part of the tooth with a thin layer of material to correct abnormalities in the color, shape, or function of the tooth. Veneer restoration can be done indirectly with porcelain material made in a laboratory and directly with composite material on the tooth surface or prefabricated which is available from the factory. Componeer is a prefabricated composite veneer that combines the aesthetic properties of ceramic veneers and the adhesive ability of composite veneers to the tooth structure. This case report describes the treatment of two central incisors that had been filled with composite and peg shapes on both lateral incisors using a componeer.

**Case report:**

A 32-year-old female patient came to the Dental Conservation Clinic at Dentistry Hospital, Padjadjaran University with the main complaint of her right and left upper front teeth and wanted to repair her old fillings and close the gap between her right and left upper front teeth and her canine teeth. Clinical examination showed that teeth 11 and 21 had been filled with composite which had changed color and had an inharmonious shape as well as a gap between the upper front teeth on the right and left sides and the right and left canine teeth.

**Treatment:**

The maxilla and mandibular teeth are molded for study models and working models. In the working model, a wax-up is carried out, then a mock-up on the patient’s teeth. Next, choose the color and size of the components that match the mock up results. Teeth 11 and 21 had their old composite fillings cleaned and refilled with dentin colored composite, teeth 13, 12, 11, 21, 22, and 23 were prepared with a minimum thickness of 0.3 mm to make room for the componeer material. The teeth was etched and bonded, and bonding was applied to the inner surface of the componeer. The composite is placed on the inner surface of the componeer then placed on the labial surface of the tooth and pressed with a special tool, then light cured. The final step is polishing.

**Treatment results:**

Teeth 13, 12, 11, 21, 22, and 23 which had undergone veneer treatment using componeer, were controlled after 1 week of treatment. The patient did not complain about the results of the treatment and said he was satisfied with the treatment.

## Introduction

Appearance is very important to gain someone’s self-confidence. The condition of the teeth greatly affects a person’s appearance, especially the anterior teeth. Aesthetic dentistry has become popular and deals with maintain and enhancement of one’s smile, while also playing a major role in the correction of facial profiles and jaw discrepancies. Aesthetic dentistry focuses primarily on the anterior region of the mouth and any imperfections in this area are of major concerns to the patient [1]. 

Dental abnormalities or anomalies such as changes in shape, size, position, color, or texture of anterior teeth will disrupt the harmony of a person’s smile. Dental anomalies can occur as a result of genetics, such as conditions in the prenatal and postnatal periods and environmental factors. Conditions during the prenatal period are the most influential in the occurrence of dental anomalies [2]. Apart from that, caries can also damage the teeth and result in loss of structure and changes in tooth color. Some of these conditions will affect aesthetics.

Maxillary lateral incisors shows variation in size, shape and form next to third molars. It is considered a developmental anomaly if the variation is too great. A peg lateral is defined as an undersized, tapered, maxillary lateral incisor which may be associated with other dental anomalies, such as canine transposition and retained deciduous teeth [3]. Peg-shaped lateral incisors are a dental anomaly that is often associated with certain gene defects and strong hereditary genes. Peg-shaped lateral incisors experience a reduction in their mesiodistal and incisal-cervical sizes. Peg-shaped teeth are usually found in lateral incisors, with a prevalence of 0.8 − 8.4%. The highest prevalence is in people of the Mongoloid race, orthodontic patients, and women [2]. 

Aesthetic dentistry and restoration using minimally invasive techniques is currently an interesting topic, thereby increasing the demand for aesthetic restorative treatment, especially for anterior teeth. The development of materials and adhesive systems makes it possible to treat aesthetic restorations with minimally invasive techniques [4]. Veneer is a minimally invasive technique for anterior teeth where there is requirement for aesthetic correction of labial surface [5]. A veneer is a layer of material placed on the labial part of the tooth to correct abnormalities in the color, shape, or function of the tooth. Veneer restoration can be done indirectly with porcelain material made in a laboratory and directly with composite material on the tooth surface or prefabricated which is available from the factory. Componeer is a prefabricated composite veneer that combines the aesthetic properties of ceramic veneers and the adhesive ability of composite veneers to tooth structure [6–8]. 

Componeer is an innovative polymerized anterior tooth restoration system that combines the advantages of direct composite restorations and veneers prefabricated. Componeer veneers (Coltene, Whaledent, Altstätten, Switzerland) are made from pre-made nanohybrid composites in the factory, have a shiny surface on the buccal area, require minimal preparation, are not sensitive, one visit and cost-effective. The thickness of the compound is 0.3 mm at the cervical area and 0.6 mm at the incisal, so only a few layers of the tooth are prepared and provide natural results [5]. 

This paper will discuss the stages of work in the treatment of aesthetic restoration of teeth that have caries on the central incisors and have a peg-shaped on the lateral incisors with direct veneers using componeer.

## Case report

**Fig. 1:**
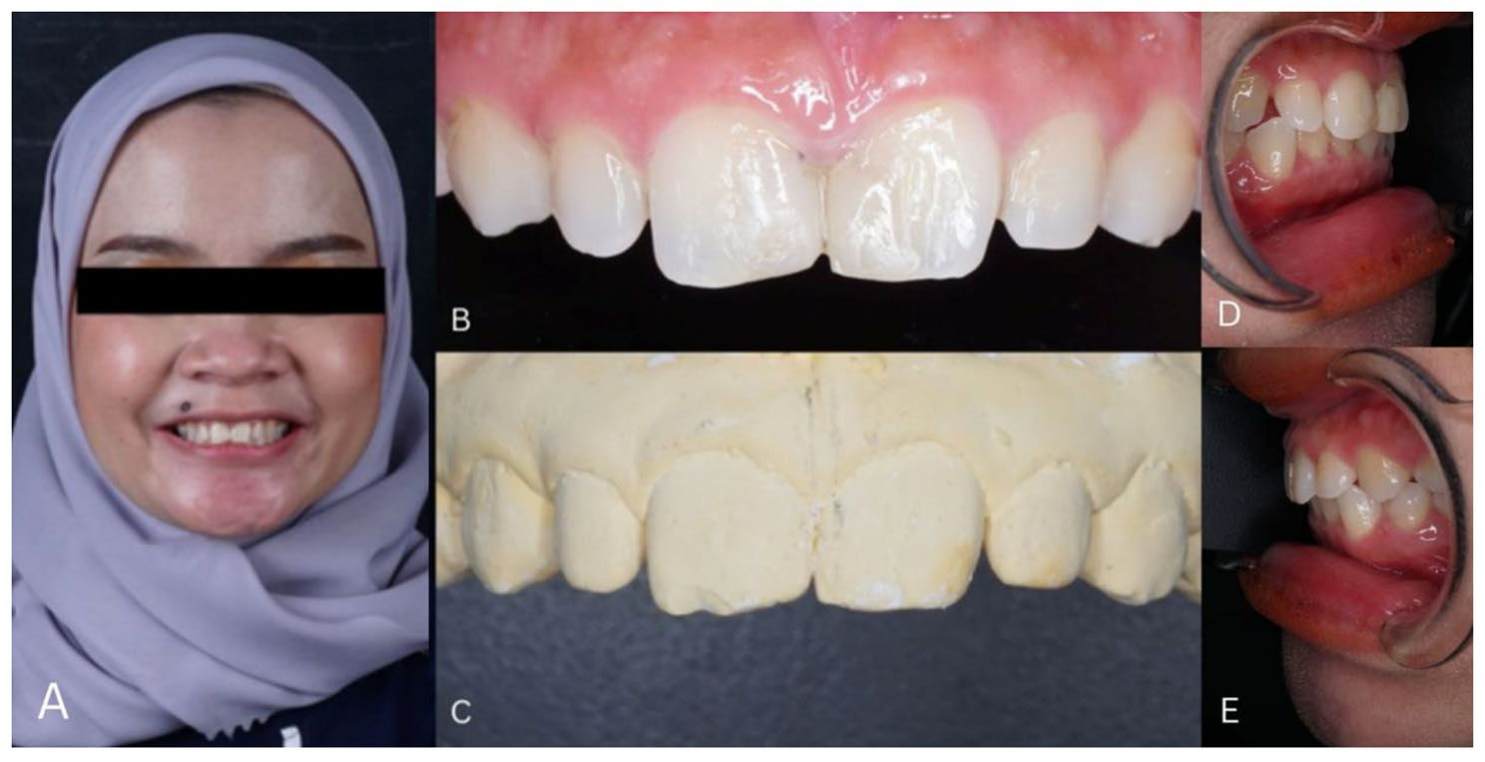
**(A)** Initial clinical condition of the patient, **(B)** Extraoral photo of the patient, **(C) **Results of the initial impression of the patient’s teeth, **(D)** Left View, **(E)** Right View

A 32-year-old female patient came to the Conservation Dentistry clinic at Dentistry Hospital, Padjadjaran University with the main complaint of her right and left upper front teeth and wanted to repair her old fillings and close the gap between her right and left upper front teeth and her canine teeth. Clinical examination showed that teeth 11 and 21 had discolored ol restorations and had an inharmonious shape. Peg shape in teeth 12 and 22, a gap between teeth 13, 12, 11, 21, 22, and 23. Pulp vitality examination showed tooth 13, 12, 11, 21, 22, and 23 is still vital. The clinical condition of teeth 12 and 22 is not in harmony with teeth 11, 21, and 13, 23. The patient’s dental history, teeth 11 and 21 had been filled with composite about 4 years ago. The patient’s medical history has no systemic diseases. In general, the condition of the patient’s dentition appears to be smaller than the normal size of the average dentition as seen in Fig. 1.

### Management

At the first visit, a complete dental examination is carried out, oral prophylaxis and making an impression for the study model and working model, as well as taking extra-oral photos of the patient.

**Fig. 2:**
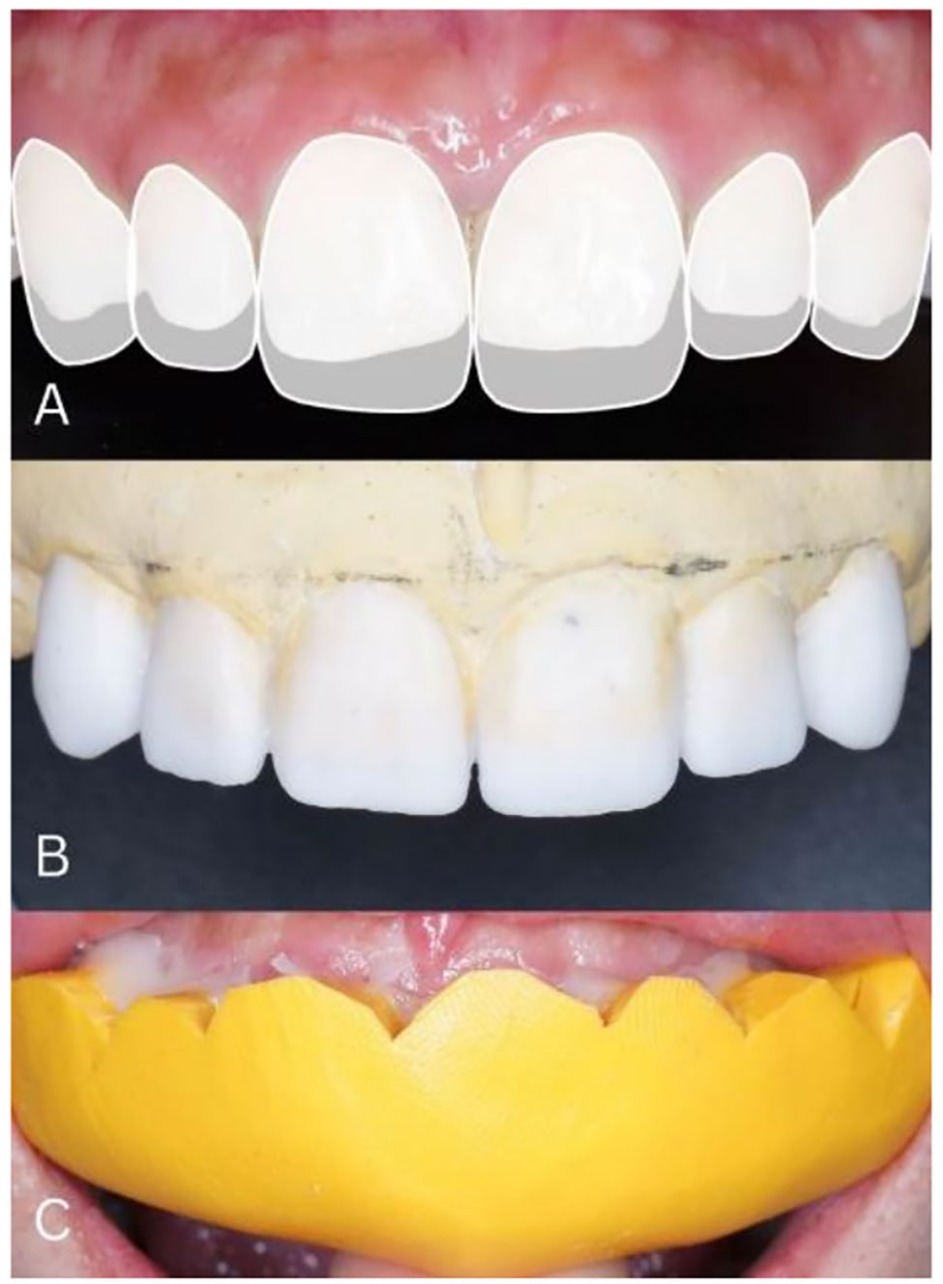
**(A)** Results of Digital Smile Design, **(B)** Wax up on the working model, **(C)** Trial of temporary veneers on a patient

Between the first and second visits, a digital smile design was carried out using Microsoft Power Point. In digital smile design, the midline of the face, the midline of the teeth, the shape of the face, the interpupillary line, the horizontal line on the incisal edge of the teeth 12, 11, 21, and 22, and the cervical borderline 11 and 21 are determined. Next, a smile analysis is carried out to see the harmony of the shape and position of the teeth with the patient’s smile lines. Teeth 13, 12, 11, 21, 22, and 23 determined ideal proportions by measuring the mesiodistal width of the tooth compared to the cervical incisal size. The ideal proportion of anterior teeth is 75 − 85%. The results of the digital smile design are used to determine the final design of the anterior dental restoration and are followed by wax-up of the working model as seen in Fig. 2.

At the second visit, the results of the digital smile design and wax-up are shown to the patient, and the patient’s consent is asked for a trial of temporary veneers. Patients are instructed to adapt to the shape and size of their teeth according to the results of a final restoration plan based on digital smile design analysis.

**Fig. 3:**
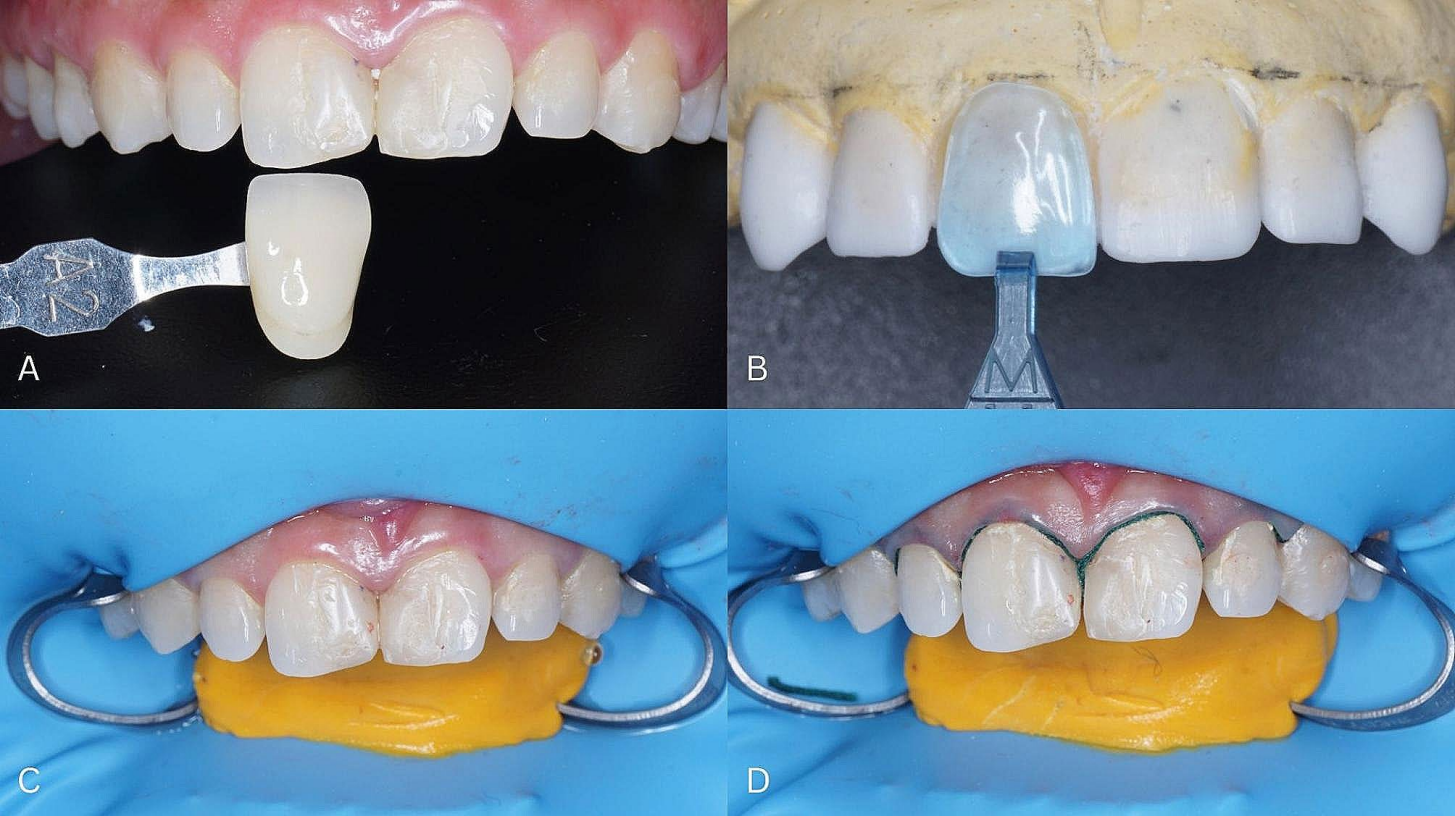
**(A)** Color selection, **(B)** Determination of componeer size, **(C)** Installation of rubber dam, **(D)** Installation of retraction cord

**Figs. 4:**
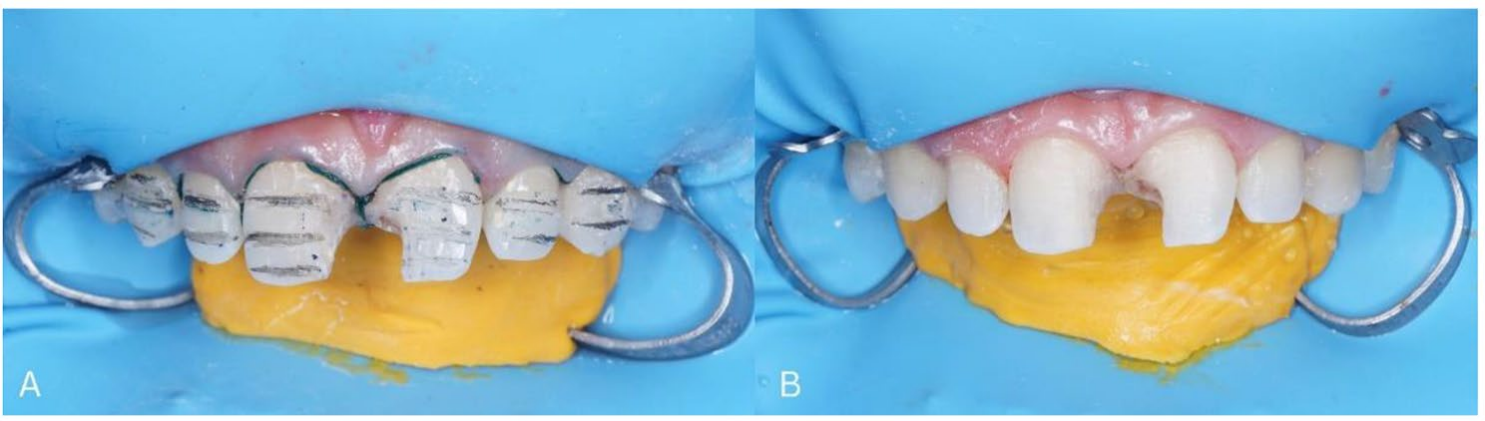
Preparation of the labial surface of the tooth

**Fig. 5:**
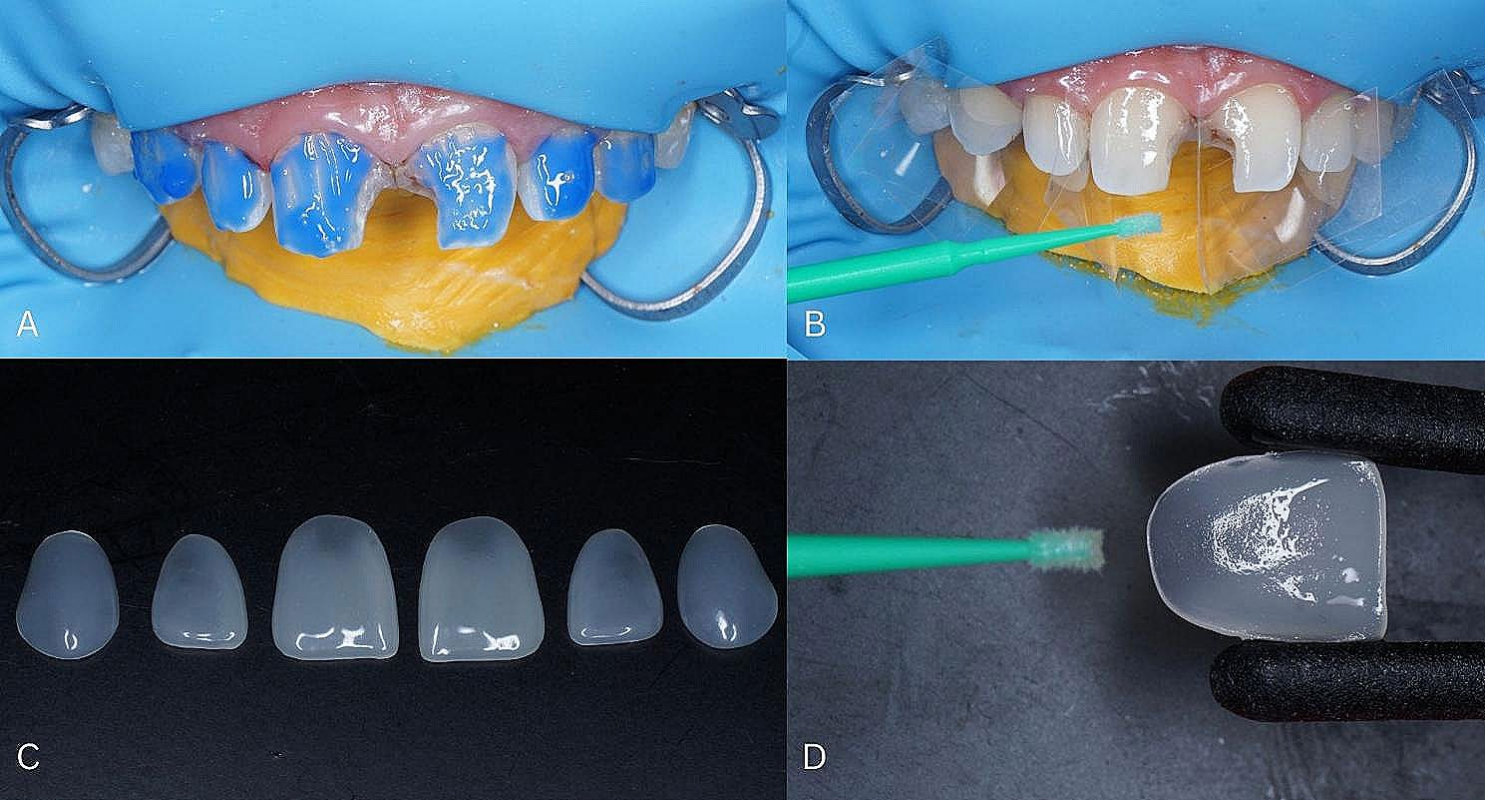
**(A)** Etching the labial surface of the tooth, **(B)** Bonding on the labial teeth, **(C)** Componeer, **(D)** Bonding on the inner surface of the componeer

**Fig. 6:**
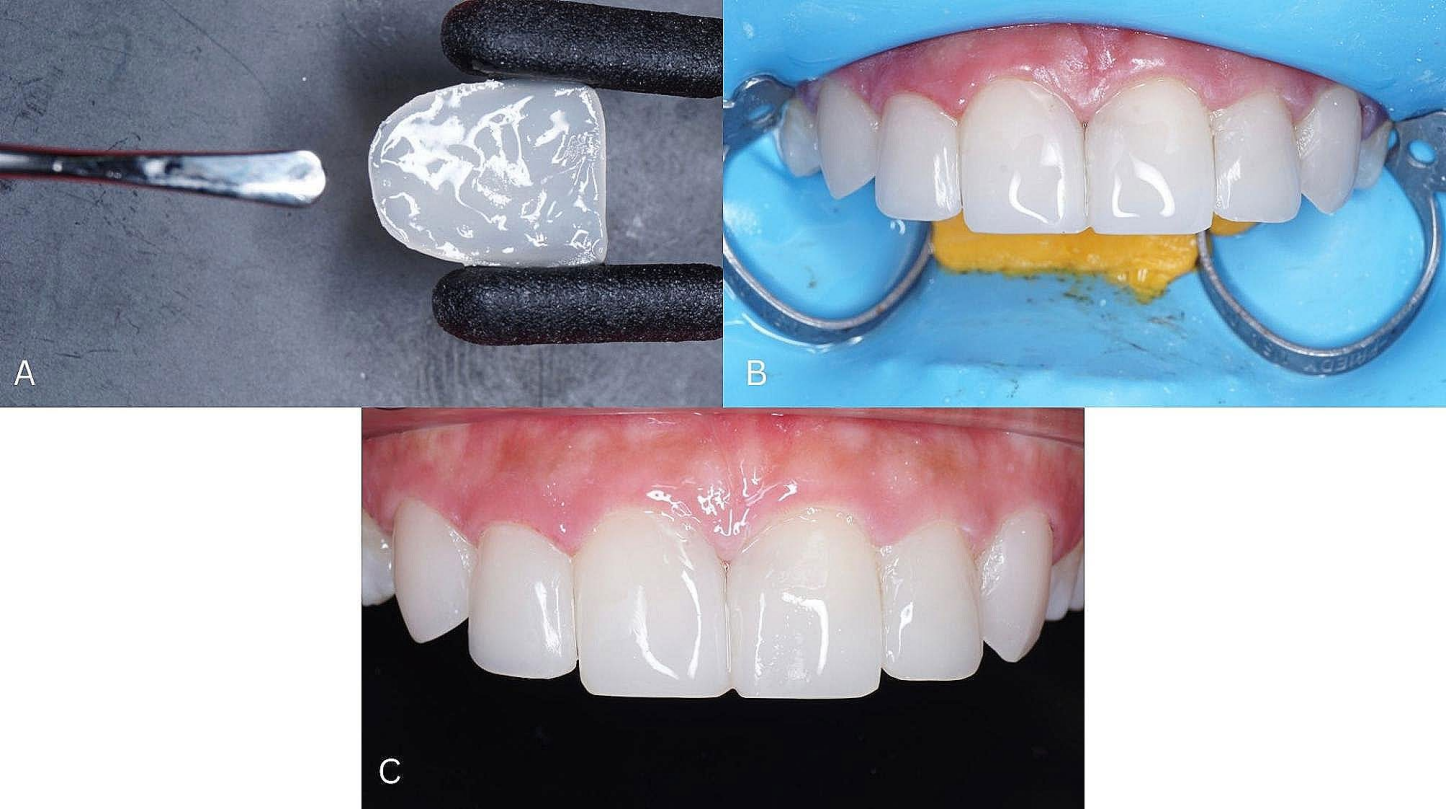
**(A)** Packable composite placed on the inner surface of the componeer, **(B)** Componeer after irradiation, **(C)** Clinical componeer after finishing and polishing

Stages of anterior tooth restoration treatment using componeer, which is:

1. Select the color of the tooth shade guide Vitapan Classical (**VITA Zahnfabrik**, H. Rauter GmbH & Co. KG, Bad Säckingen) that will be used to restore the tooth 13, 12, 11, 21, 22, and 23 (Fig. 3A).
2. Determine the size of the componeer (Coltene, Whaledent, Altstätten, Switzerland) using the template, holding it close to the wax-up result (Fig. 3B).
3. Anesthesia (Orabloc, Pharos, Capua, Italy) in the anterior region of the maxilla.
4. Installation of rubber dam (Sanctuary, Malaysia, Sanctuary Dental by Sanctuary Health Sdn Bhd) using split dam technique (Fig. 3C).
5. Installation of retraction cord (Sure Endo, South Korea, Sure Dent Corp.) in dental sulcus 13, 12, 11, 21, 22, and 23 (Fig. 3D).

6. Prepare teeth 11 and 21 by removing all previous restorations and the labial surface of tooth 13, 12, 11, 21, 22, and 23 to a depth of 0.3 mm (Fig. 4A and B).

7. Etch the entire labial surface of teeth 13, 12, 11, 21, 22, and 23 using 37% phosphoric acid (Total Etch, Ivoclar-Vivadent) (Fig. 5A).
8. Bonding (One Bond Coat, Coltene, Altstatten, Switzerland) the labial surface of teeth 13, 12, 11, 21, 22, and 23 and the inner surface of the componeer (Fig. 5B, C and D).

9. Place the packable composite (Coltene, Whaledent, Altstätten, Switzerland) according to the specified color on the inner surface of the componeer and smooth it (Fig. 6A).
10. Place the componeer (Coltene, Whaledent, Altstätten, Switzerland) on the labial surface of the tooth, then press it with the placement tool, make sure the entire labial surface of the tooth is covered with componeer.
11. Irradiate (Curing Pen, Eighteeth, Changzhou Sifary Medical Technology Co.,Ltd, China) the labial and palatal surfaces of teeth 13, 12, 11, 21, 22, and 23 (Fig. 6B).
12. Perform finishing and polishing on the restoration surface (3 M-ESPE, St Paul, MN, USA) (Fig. 6C).

The final restoration results show an increase in the height and width of each tooth to achieve a golden proportion of 80% (i.e. tooth 11 height = 10 mm and width = 8 mm, teeth 12 and 22 height = 7.5 mm and width = 6 mm, teeth 13 and 23 height = 8.75 mm and width 7 mm).

**Fig. 7:**
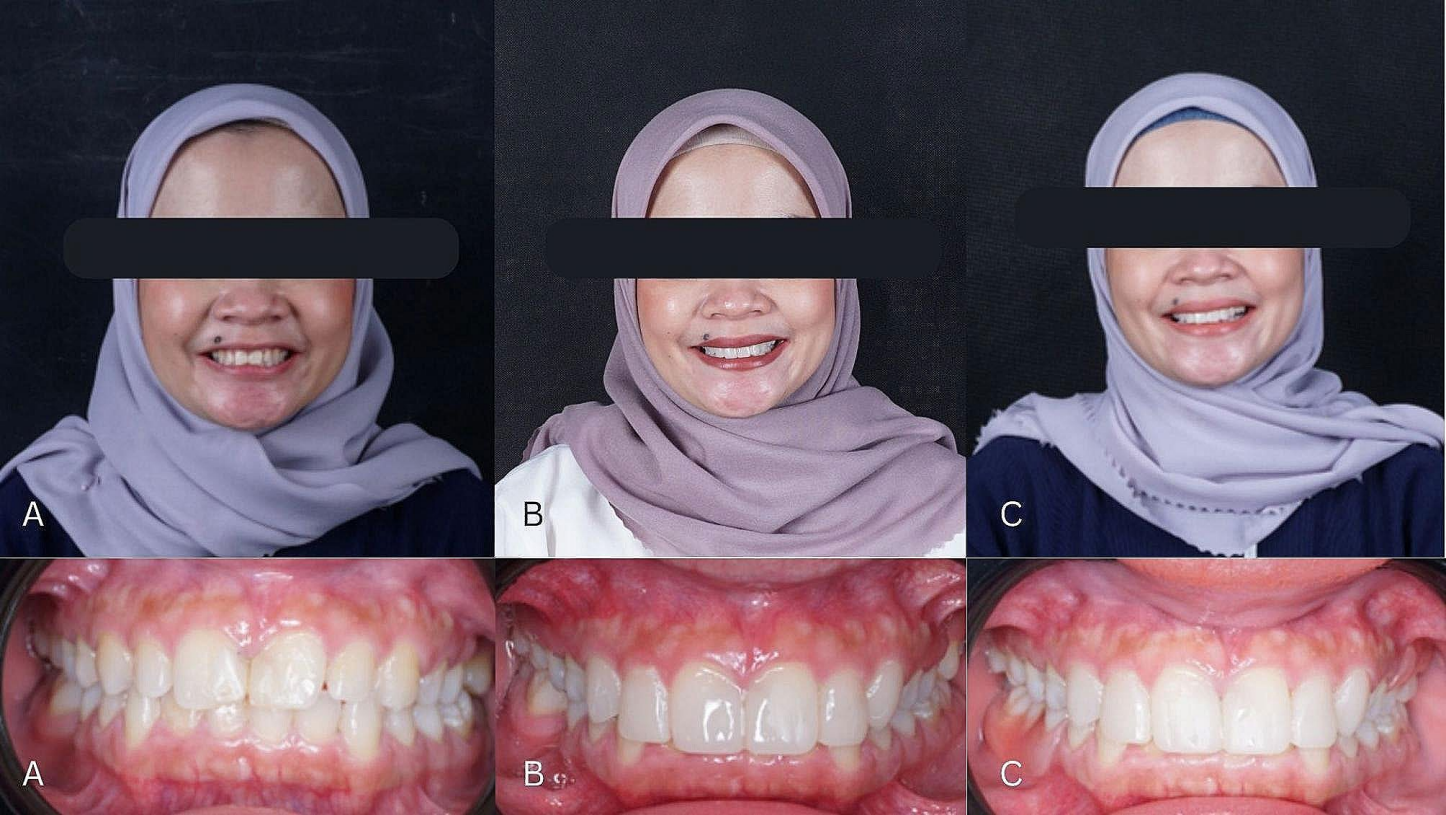
**(A)** Extraoral and Intraoral photo before treatment, **(B)** Extraoral and Intraoral photo after 1 week, **(C)** Extraoral and Intraoral photo after 1 month

Restoration evaluation was carried out after 1 week of treatment as seen in Fig. 7. The patient did not complain about the results of the restoration and expressed satisfaction with the treatment of his front teeth.

## Discussion

Today, the development of the times has had an impact on changes in people’s lifestyles. People prioritize appearance to support their professional and personal lives. Therefore, many patients come to the dentist to correct the shape of their teeth to achieve good aesthetics and a more harmonious smile. Damage to the shape of the teeth due to caries or dental anomalies in the anterior teeth that are present at birth are the cause of a person’s disharmonious smile [1]. 

Anomalies in tooth number, structure, and morphology can occur in permanent teeth due to genetic and epigenetic influences. Clinical manifestations of dental anomalies include microdontia, macrodontia, hypodontia and oligodontia. Koch et al. defined abnormal tooth size as a state of dimensional deviation of two standard deviations from the average. Size anomalies manifest clinically in the form of macrodontia and microdontia [2]. Microdontia is a rare phenomenon. The term microdontia (microdentism, microdontism) is defined as the condition of having abnormally small teeth. According to Boyle, “in general microdontia, the teeth are small, the crowns short, and normal contact areas between the teeth are frequently missing” [9]. 

Microdontia is a dental condition in which a tooth appears to be smaller than the normal tooth size [2]. Generally microdontia occurs in permanent teeth, rarely in primary teeth. Microdontia has two types, which are type 1 microdontia (true microdontia) and type 2 (pseudo microdontia). True microdontia is a tooth size that is smaller than normal in a normal-sized jaw, while pseudo microdontia is all normal teeth size but appears smaller relative to a large jaw. Based on the number of teeth affected, microdontia can be classified as localized microdontia and generalized microdontia. Localized microdontia only involves 1 or 2 teeth, while generalized microdontia involves all teeth. True generalized microdontia is the condition of all normally shaped teeth with a size smaller than the normal average tooth size, while generalized relative microdontia is all small teeth in a large jaw. The etiology of microdontia is multifactorial, but the main causes are genetics and growth and development disorders [10]. The initiating factor or factors responsible for microdontia remain obscure. Genetic factors probably play a role in the formation of microdontia [9]. 

Microdontia occurs due to deviations at the beginning of tooth growth and development, namely at the bud stage of the 8th intrauterine week. Deviations in tooth development result in ameloblasts and odontoblasts as tooth-forming cells not differentiating optimally, resulting in teeth that are smaller than normal. The main factors that influence dental anomalies are genetic and environmental. Genetic factors influence tooth germs through genes inherited from parents, while environmental factors influence teeth after eruption, such as mechanical and chemical factors [10]. Microdontia can also be an oral manifestation of several syndromes, such as Down Syndrome, ectodermal dysplasia, Silver-Russel Syndrome, William Syndrome, Gorlin- Chaundhry-Moss Syndrome [2]. 

Anomaly tooth shape can be managed by carrying out restoration treatment, which can be done directly or indirectly. Direct restoration can be carried out using composite materials or using prefabricated materials known as componeer. Composite veneers are available in a wide choice of colors and opacity, so they can mimic the natural color of dentin and enamel. Componeers are manufactured from nanohybrid composite that ensures excellent homogeneity and stability of the enamel shells. Componeer (Coltene, Altstatten, Switzerland) prefabricated veneers are thin composite resin shells (0.3 mm cervically and 0.6-1.0 mm to the incisal edge), made of a pre-polymerized hybrid composite resin, synergy D6 (Coltene). The veneers are cemented with the same hybrid composite resin that they are made from, which has the potential of making the complete restoration as a monoblock unit. The extremely thin veneer (0.3 mm) allows conservation of tooth structure. The micro-retentive inner surface ensures a last bond, therefore, conditioning of the veneer is not required, making it a milestone in veneers. This Componeer treatment is operator friendly, minimally invasive and single appointment procedure [5, 11]. One of the advantages of direct veneers is that the patient’s visit for treatment is shorter compared to indirect veneers [12]. 

The concept of direct veneer using prefabricated has been developing since the early 1980s, using acrylic known as the Mastique Laminate Veneer System (Caulk, Milford, DE, USA). The intaglio surface of the Mastique Veneer is etched using polyacrylic acid and then the veneer is adapted to the labial surface of the tooth which has been etched using composite and bonding without filler. Mastique veneer has many disadvantages due to technological limitations and poor veneer surface quality. As material technology and adhesives continue to develop, prefabricated veneers made from composites, known as componeers, have developed [12]. 

Componeer (Coltene, Whaledent) is made from a nanohybrid composite which provides optimal aesthetic, functional, and economical results and is fast to use. Componeer is available in various sizes and has two color choices, which are transparent and bleaching. Choosing the appropriate dentin color under the componeer will give the componeer a natural color. Componeer that are damaged are easier to correct or repair [4, 13, 14]. 

Based on clinical results and statistical analysis from Parag Dua et al., the study concluded that both “componeers” and direct composite veneers showed minimal changes in color, surface texture, and marginal integrity and displayed excellent gingival response. The gingival responses improved over the period of study. Componeers present a conservative veneering modality and remarkable advancement due to superior esthetics and monobloc properties [13, 14]. Componeers are resin material similar to composite resins used in dentistry. They are thin shells of precured resins; unlike porcelain which contains silica and is similar to glass. Its mechanical strength is much lower than that of porcelain and surface hardness is lesser than porcelain. Porcelain is highly likely to break and get crushed whereas componeers are generally unbreakable. Porcelain has higher chance of chipping off than componeers. Similarly, though the surface hardness in porcelain is more, however, the polishability of porcelain is higher with the result of a glossy appearance. For porcelain veneers or laminates, at least 2 to 3 mm of tooth surface would have to be reduced as the porcelain themselves are about 1 mm thick, whereas componeer only need 0,3 mm to 0,6 mm labial reduce thickness [15]. 

Even though porcelain is brilliant glossy with a permanent finish, since it is expensive, it cannot be afforded by many. Patients should have different treatment options. Componeers may be the solution to an average working class population. They have considerable advantages for the dentist such as easy and efficient to use, only one session required, quality dental restorations with excellent aesthetic results, no impressions or laboratory necessary, optimum customization (choice of colour, highlighting shape and structure), economical for dentists and patients due to high success, rate and efficiency, and they can be repaired intraorally in one session [15, 16]. 

## Conclusion

One of the aesthetic restoration treatments to correct teeth damaged by caries or tooth shape anomalies is veneer treatment. Veneers can be done directly or indirectly. Direct veneers can be done by placing the composite directly on the labial surface of the tooth or prefabricated. Prefabricated veneers (componeer) have several advantages over indirect veneers, which are they can be placed directly on the patient’s teeth in one visits, more conservative and can use to restore the entire front arc, without going through the manufacturing process in a laboratory. In addition, the componeer restoration can be costumized to size, shape and texture just before insertion and the cost to the patient is more economical. This treatment need follow up over time to verify their stability over time, both in shape and colour.

## Data Availability

Data and materials ability should be contacted to correspondence author: irmaleny@unpad.ac.id.
